# Supplementary material for: Translating transcriptomic findings from cancer model systems to humans through joint dimension reduction
Source: Commun Biol. 2023 Feb 16;6:179. doi: 10.1038/s42003-023-04529-3 (PMC9935626; doi:10.1038/s42003-023-04529-3)
Supplement: Supplementary file 2 — Supplementary Information [file 42003_2023_4529_MOESM2_ESM.pdf]

Supplemental Figure S1.

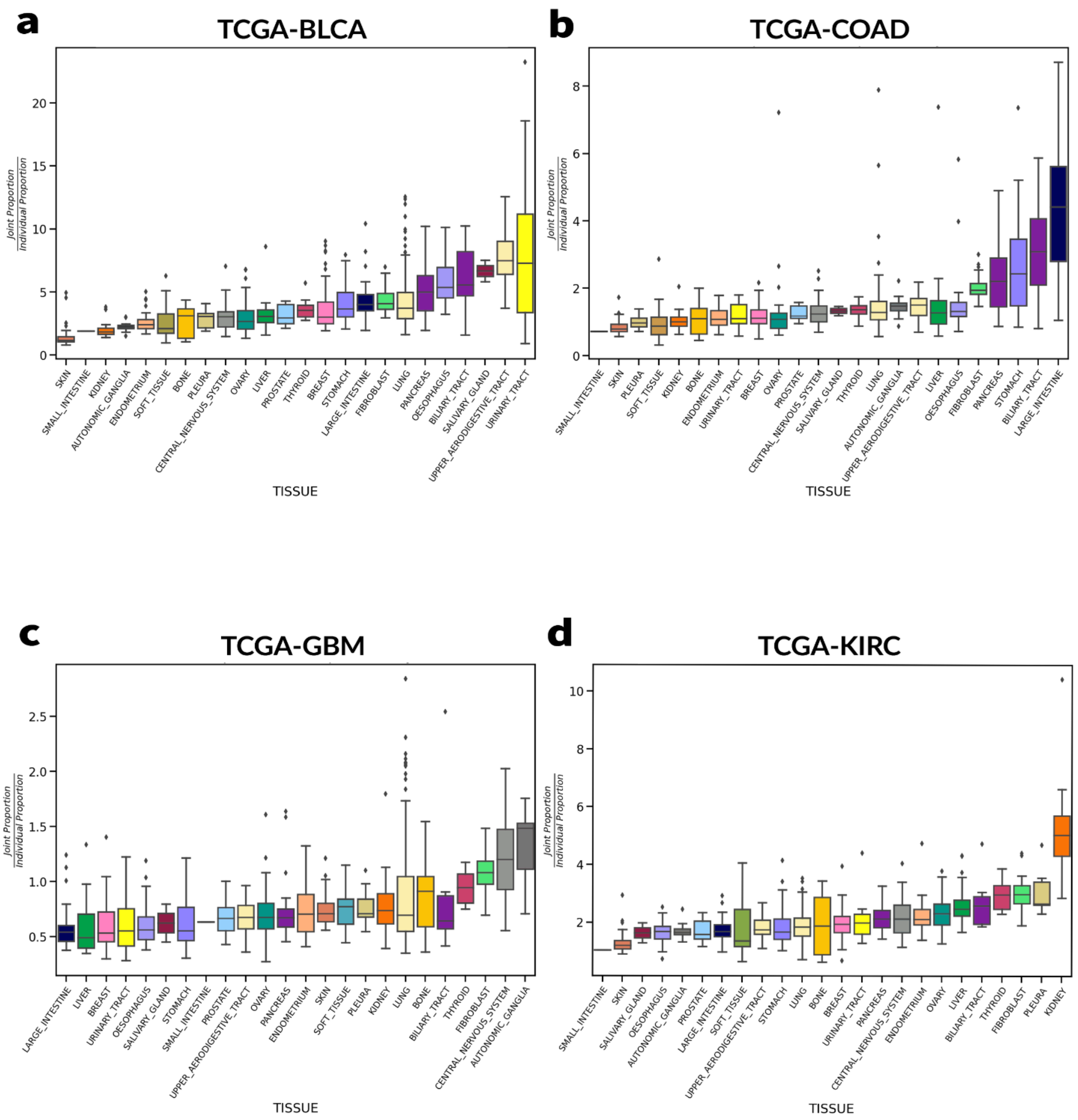

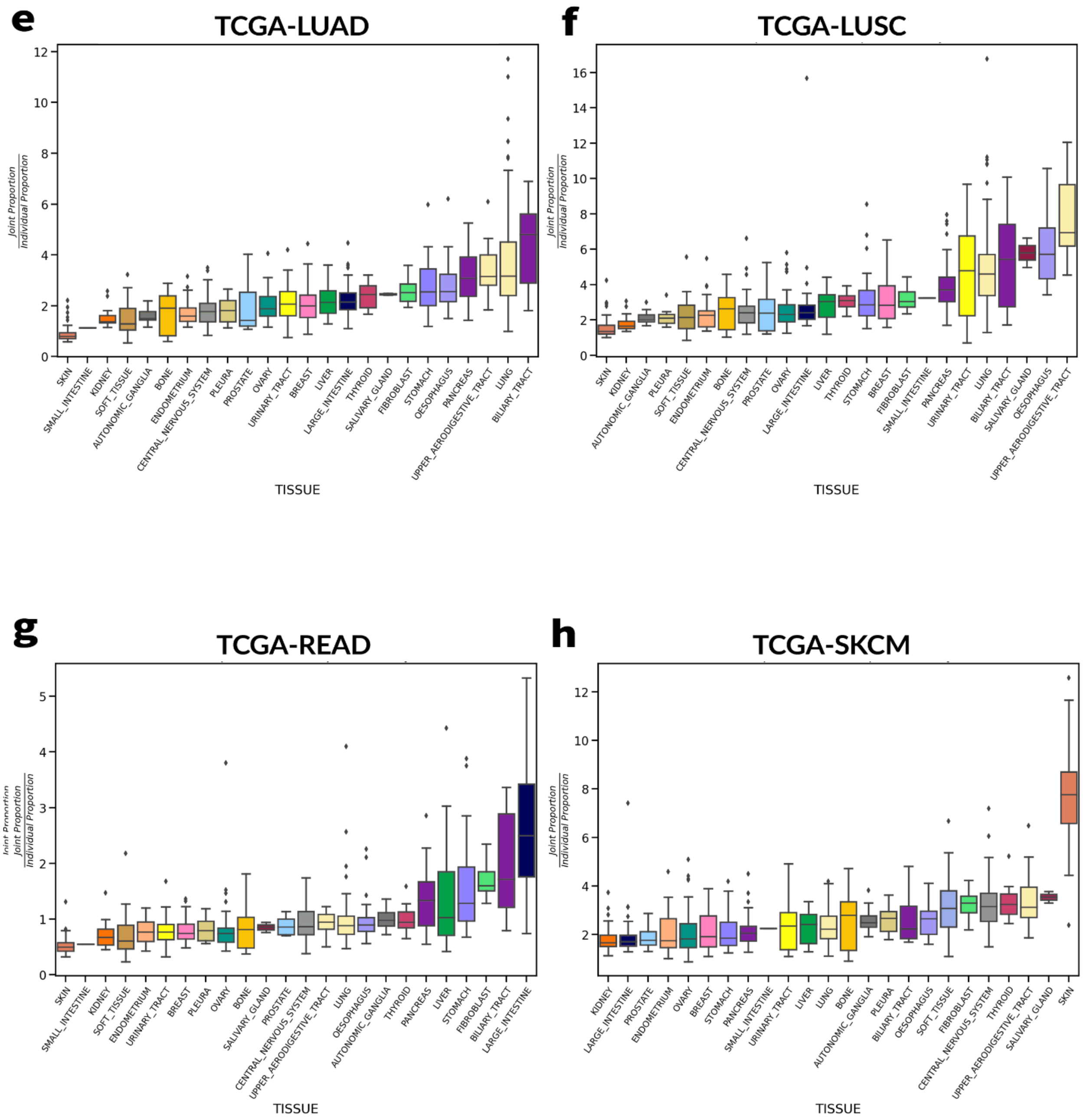

i

	Number of cell lines		
LUNG	173	LIVER	25
LARGE_INTESTINE	54	URINARY_TRACT	25
BREAST	50	KIDNEY	23
CENTRAL_NERVOUS_SYSTEM	49	SOFT_TISSUE	17
SKIN	47	BONE	16
OVARY	45	AUTONOMIC_GANGLIA	15
PANCREAS	41	THYROID	11
STOMACH	37	PLEURA	9
FIBROBLAST	34	BILIARY_TRACT	7
UPPER_AERODIGESTIVE_TRACT	30	PROSTATE	7
ENDOMETRIUM	28	SALIVARY_GLAND	2
OESOPHAGUS	27	SMALL_INTESTINE	1

Supplemental Figure S1. Joint dimension reduction integration of CCLE with TCGA subtypes. (**a-h**) Boxplots of best represented cell line groups according to joint/individual proportion metric. (**a**) Urothelial Bladder Carcinoma, TCGA-BLCA (**b**) Colon Adenocarcinoma, TCGA-COAD (**c**) Glioblastoma Multiforme, TCGA-GBM (**d**) Kidney Renal Clear Cell Carcinoma, TCGA-KIRC (**e**) Lung Adenocarcinoma, TCGA-LUAD (**f**) Lung Squamous Cell Carcinoma, TCGA-LUSC (**g**) Rectum Adenocarcinoma, TCGA-READ (**h**) Cutaneous Melanoma, TCGA-SKCM (**i**) Table describing the number of cell lines for each tissue type in CCLE.

Supplemental Figure S2.

a

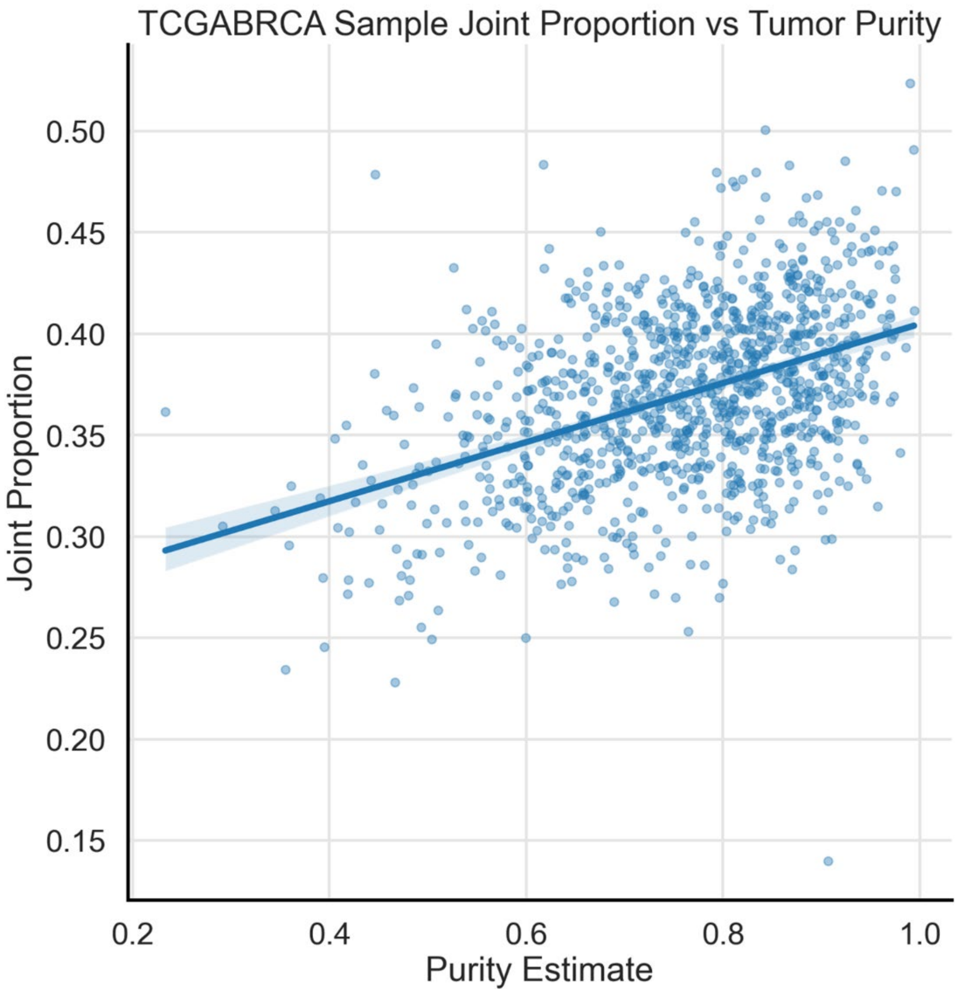

Supplemental Figure S2. Joint variation and tumor purity. (**a**) Scatterplot relating TCGA-BRCA consensus tumor purity measurements to the magnitude of joint variation was determined through AJIVE when integrated with CCLE.[31]

Supplemental Figure S3.

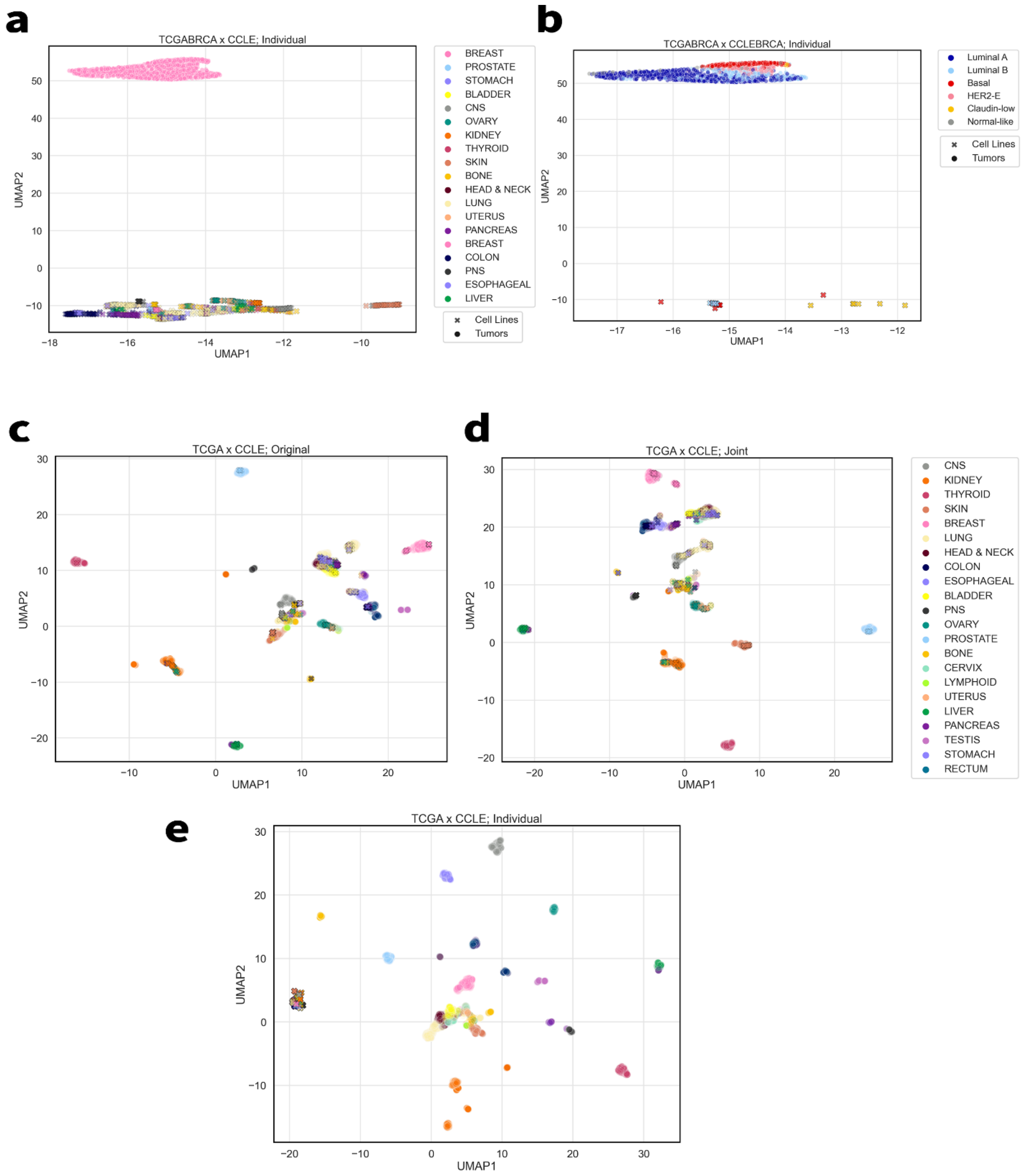

Supplemental Figure S3. UMAP clustering of joint dimension reduced integrated datasets. (**a-b**) 2D projections of combined cell line and breast tumor individual projection matrices using UMAP. (**c-e**) Clustering of samples when integrating pan-cancer TCGA (n = 10,305) and CCLE (n = 935) colored by tissue type.

Supplemental Figure S4.

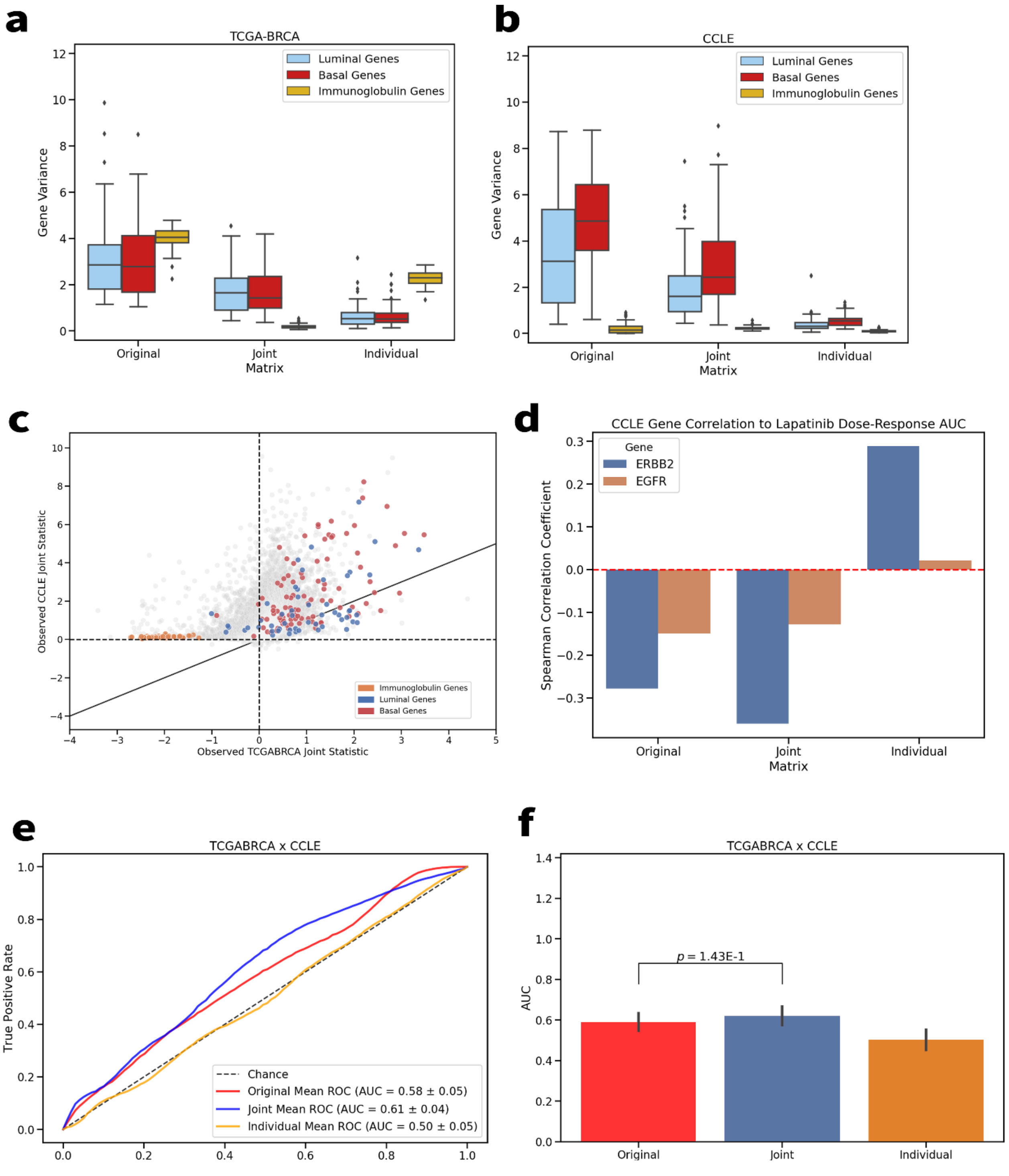

Supplemental Figure S4. Joint dimension reduction integration of CCLE with TCGA-BRCA. (**a-b**) Boxplots of gene variances of published luminal, basal, and immunoglobulin activity gene sets grouped by Original, Joint, and Individual projection matrices. (**c**) Scatterplot comparing joint statistics for each gene with gene sets highlighted. (**d**) Spearman correlation coefficients of ERBB2 and EGFR expression to lapatinib dose-response AUC in CCLE. (**e**) Receiver operating characteristic (ROC) curve of elastic net ERBB2-targeting response models trained from Original, Joint, and Individual CCLE AJIVE projection matrices. Models were tested on CALGB 40601 using pathological complete response (pCR) as the positive class. (**f**) Barplot of mean ROC-AUCs. Emprirical p-values were derived from differences between bootstrapped predictions. Error bars represent 95% confidence intervals.

Supplemental Figure S5.

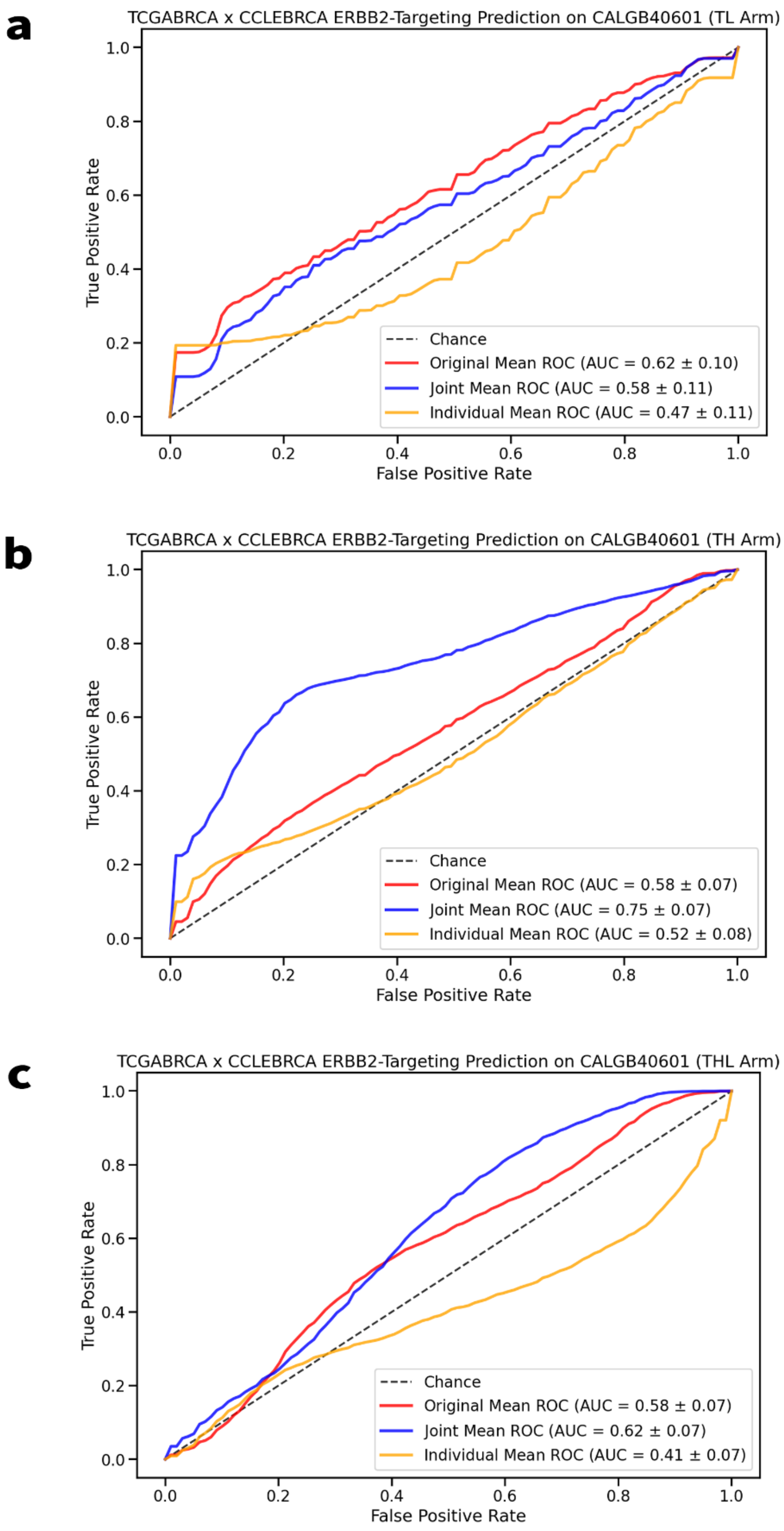

Supplemental Figure S5. CALGB 40601 predictions by clinical trial arm. (**a**) ROC curves of CCLE-BRCA trained ERBB2-targeting model on CALGB 40601 paclitaxel+lapatinib (TL) arm (**b**) paclitaxel+trastuzumab (TH) arm. (**c**) paclitaxel+lapatinib+trastuzumab (THL) arm

Supplemental Figure S6.

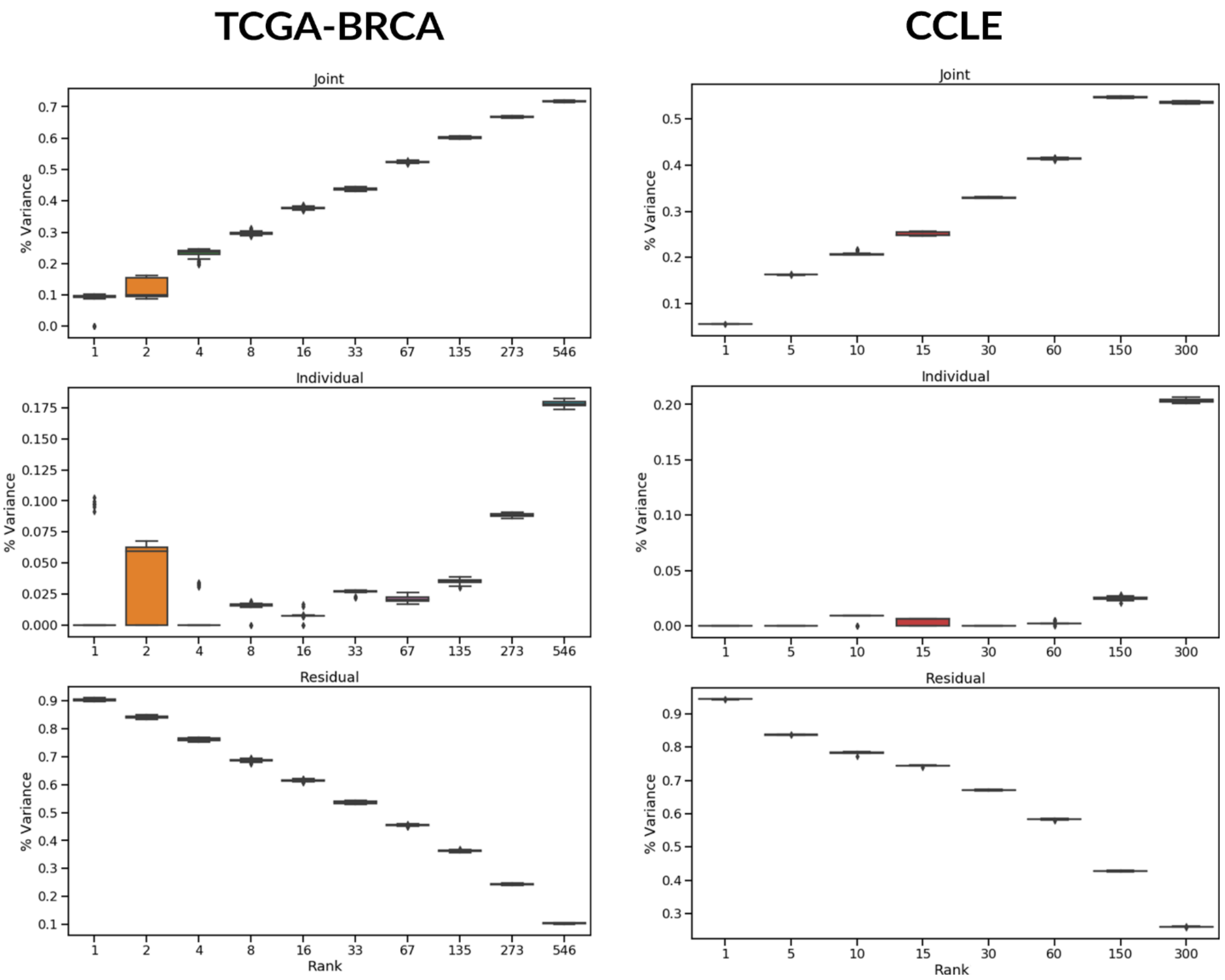

Supplemental Figure S6. Initial AJIVE rank selection through bootstrapping. Boxplots of joint variation percent by rank selection. The individual point of inflection marks the rank at which joint variation is maximized and individual variation in minimized.

Supplemental Figure S7.

a

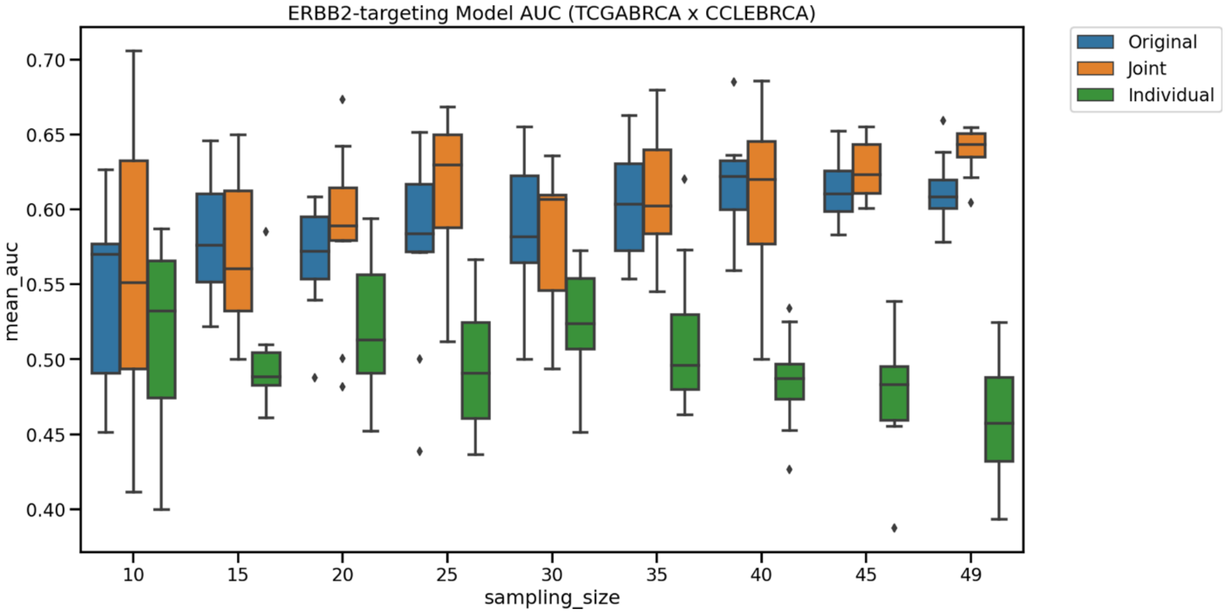

Supplemental Figure S7. Bootstrap simulation applying jDR integration and predictive model training at lower sample sizes. (**a**) Boxplots of mean ROC-AUCs of ERBB2-targeting response predictive models trained from CCLE-BRCA. Breast cell lines were randomly selected without replacement and integrated with TCGA-BRCA. Linear elastic net models were trained from the selected breast cell lines. The procedure was repeated 50 times at each size.
