# Supplementary material for: Translating transcriptomic findings from cancer model systems to humans through joint dimension reduction
Source: Commun Biol. 2023 Feb 16;6:179. doi: 10.1038/s42003-023-04529-3 (PMC9935626; doi:10.1038/s42003-023-04529-3)
Supplement: Supplementary file 3 — Reporting Summary-New [file 42003_2023_4529_MOESM3_ESM.pdf]

nature portfolio

Corresponding author(s): Joel S. Parker, PhD

Last updated by author(s): Nov 10, 2022

Reporting Summary

Nature Portfolio wishes to improve the reproducibility of the work that we publish. This form provides structure for consistency and transparency in reporting. For further information on Nature Portfolio policies, see our Editorial Policies and the Editorial Policy Checklist.

Statistics

For all statistical analyses, confirm that the following items are present in the figure legend, table legend, main text, or Methods section.

n/a	Confirmed	
☐	☒	The exact sample size (*n*) for each experimental group/condition, given as a discrete number and unit of measurement
☐	☒	A statement on whether measurements were taken from distinct samples or whether the same sample was measured repeatedly
☐	☒	The statistical test(s) used AND whether they are one- or two-sided<br>*Only common tests should be described solely by name; describe more complex techniques in the Methods section.*
☒	☐	A description of all covariates tested
☐	☒	A description of any assumptions or corrections, such as tests of normality and adjustment for multiple comparisons
☐	☒	A full description of the statistical parameters including central tendency (e.g. means) or other basic estimates (e.g. regression coefficient) AND variation (e.g. standard deviation) or associated estimates of uncertainty (e.g. confidence intervals)
☐	☒	For null hypothesis testing, the test statistic (e.g. *F*, *t*, *r*) with confidence intervals, effect sizes, degrees of freedom and *P* value noted<br>*Give P values as exact values whenever suitable.*
☒	☐	For Bayesian analysis, information on the choice of priors and Markov chain Monte Carlo settings
☒	☐	For hierarchical and complex designs, identification of the appropriate level for tests and full reporting of outcomes
☐	☒	Estimates of effect sizes (e.g. Cohen's *d*, Pearson's *r*), indicating how they were calculated

Our web collection on statistics for biologists contains articles on many of the points above.

Software and code

Policy information about availability of computer code

Data collection	All public data used in the analysis has been uploaded to https://webshare.bioinf.unc.edu/public/baprice/AJIVE_jDR_Integration/.
Data analysis	Step-by-step code to replicate the analyses and figures in the manuscript is available at https://github.com/baprice/AJIVE-jDR-Integration.

For manuscripts utilizing custom algorithms or software that are central to the research but not yet described in published literature, software must be made available to editors and reviewers. We strongly encourage code deposition in a community repository (e.g. GitHub). See the Nature Portfolio guidelines for submitting code & software for further information.

Data

Policy information about availability of data

All manuscripts must include a data availability statement. This statement should provide the following information, where applicable:

- Accession codes, unique identifiers, or web links for publicly available datasets
- A description of any restrictions on data availability
- For clinical datasets or third party data, please ensure that the statement adheres to our policy

TCGA-BRCA, CCLE, and mouse data used in this article before and after integration can be downloaded at https://webshare.bioinf.unc.edu/public/baprice/AJIVE_jDR_Integration/. Processed data for eight other TCGA subtypes (TCGA-BLCA, TCGA-COAD, TCGA-GBM, TCGA-KIRC, TCGA-LUAD, TCGA-LUSC, TCGA-READ, TCGA-SKCM) are also provided at the link. Remaining data are available as noted in the article or in the jupyter notebooks upon reasonable request, and according to data use guidelines of each particular data set.

Field-specific reporting

Please select the one below that is the best fit for your research. If you are not sure, read the appropriate sections before making your selection.

☒ Life sciences ☐ Behavioural & social sciences ☐ Ecological, evolutionary & environmental sciences

For a reference copy of the document with all sections, see nature.com/documents/nr-reporting-summary-flat.pdf

Life sciences study design

All studies must disclose on these points even when the disclosure is negative.

Sample size	Initial selections of TCGA-BRCA (n=1102) and CCLE (n=935) datasets were chosen due to similar sample sizes. However, we found input sample size heterogeneity to have minimal effect on integration outcomes when using AJIVE. Thus, we selected datasets based on biological relevance instead.
Data exclusions	Genes that were not present in both input datasets were excluded.
Replication	Results can be replicated through the Jupyter Notebooks found at https://github.com/baprice/AJIVE-jDR-Integration.
Randomization	Study does not involve data collection.
Blinding	Study does not involve data collection.

Reporting for specific materials, systems and methods

We require information from authors about some types of materials, experimental systems and methods used in many studies. Here, indicate whether each material, system or method listed is relevant to your study. If you are not sure if a list item applies to your research, read the appropriate section before selecting a response.

Materials & experimental systems

n/a	Involved in the study	
☒	☐	Antibodies
☒	☐	Eukaryotic cell lines
☒	☐	Palaeontology and archaeology
☒	☐	Animals and other organisms
☒	☐	Human research participants
☐	☒	Clinical data
☒	☐	Dual use research of concern

Methods

n/a	Involved in the study	
☒	☐	ChIP-seq
☒	☐	Flow cytometry
☒	☐	MRI-based neuroimaging

Clinical data

Policy information about clinical studies

All manuscripts should comply with the ICMJE guidelines for publication of clinical research and a completed CONSORT checklist must be included with all submissions.

Clinical trial registration	NCT00770809
Study protocol	*Note where the full trial protocol can be accessed OR if not available, explain why.*
Data collection	*Describe the settings and locales of data collection, noting the time periods of recruitment and data collection.*
Outcomes	*Describe how you pre-defined primary and secondary outcome measures and how you assessed these measures.*
